# Supplementary material for: Effects of Early Life Stress on Bone Homeostasis in Mice and Humans
Source: Int J Mol Sci. 2020 Sep 10;21(18):6634. doi: 10.3390/ijms21186634 (PMC7556040; doi:10.3390/ijms21186634)
Supplement: Supplementary file 1 [file ijms-21-06634-s001.pdf]

Supplementary Data

Supplementary Figures

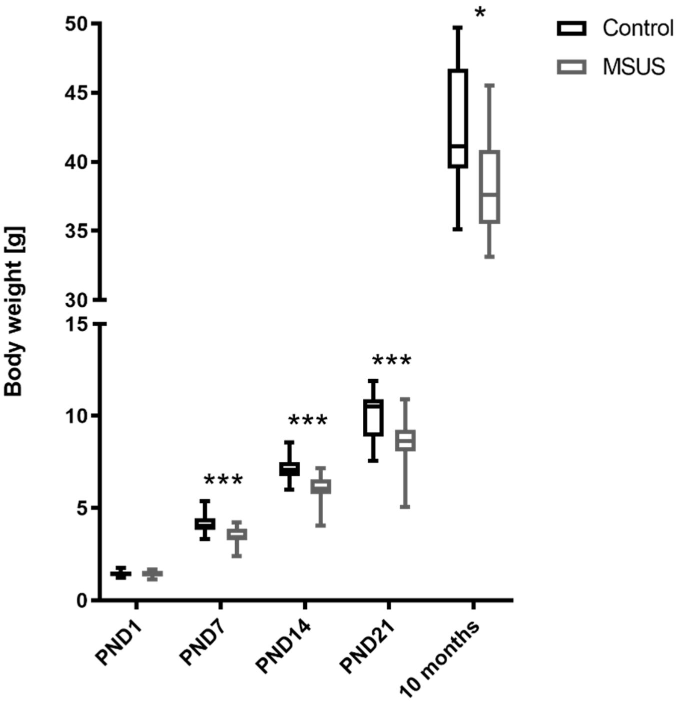

S-Figure 1: Body weight development. Shown are body weights of male pups, which are equal at PND1 and decreased following MSUS at PND7 (n = 45 Control, n = 46 MSUS), PND14 (n = 45 Control, n = 46 MSUS), PND21 (n = 46 Control, n = 44 MSUS) and in adults (n = 14 Control, n = 14 MSUS). Data are expressed as Min to Max of body weight in [g] and considered significant at $^*p < 0.05$ and $^{***}p < 0.001$.

Supplementary Tables

S-Table 1: Micro-CT data obtained for full, cortical (Ct) and trabecular (Tb) bone in control mice and MSUS mice with regard to length, total volume (TV), bone volume (BV), marrow volume (MV), apparent volume density (AVD), area (Ar), thickness (Th), bone surface (BS), spacing (Sp) and number (N) and thereof calculated characteristics.

		Control	MSUS	
	**Variables**	**Adjusted Mean**	**Adjusted Mean**	**p-value (adjusted)[1]**
**Full Bone**	Length (mm)	16.45	16.37	0.263
	TV (mm^3)	44.23	42.88	0.129
	BV (mm^3)	21.31	20.48	0.128
	MV (mm^3)	22.92	22.40	0.457
	AVD (%)	48.19	47.77	0.675
**Cortical Bone**	Tt.Ar (mm^2)	0.92	0.90	0.478
	Ct.Ar/Tt.Ar (%)	40.31	40.36	0.959
	Ct.Th (mm)	0.18	0.18	0.844
**Trabecular Bone**	TV (mm^3)	5.19	5.10	0.558
	BV/TV (%)	6.99	7.26	0.792
	MV/TV (%)	93.01	92.74	0.792
	BS/BV (mm/mm^3)	45.10	47.37	0.203
	Tb.Th (mm)	0.07	0.07	0.282
	Tb.Sp (mm)	0.49	0.44	0.319
	Tb.N (1/mm)	1.98	2.14	0.452

[1] adjusted for body weight at a covariate mean of 16.41. Significance level: all $p > 0.05$.

S-Table 2: Taqman Gene Expression Assays.

Target	Identifier
GAPDH	Mm99999915_g1
Osteocalcin	Mm03413826_mH
Osteoprotegerin	Mm00435452_m1
Osteopontin	Mm00436767_m1
Sclerostin	Mm00470479_m1

S-Table 3: Self-Designed Primers.

NCBI Accession Nr	Target Identifier / Primer Sequence
NM_007420	ADRB2
muAdrb2 - Forward	gATTgCAgTggATCgCTATgTTg
muAdrb2 - Reverse	gACCACTCgggCCTTATTCTTg
muAdrb2 -TM	F-ATCACATCgCCCTTCAAgTACCAgAgC-Q
NM_001033124	NtTRK
muNtrk1 - Forward	gggAgTTgAgAAgCCTAACCAT

muNtrk1 - Reverse	CgCATTggAggACAgATTCA
muNtrk1 -TM	F-CAgATgCCTTCCgTTTCACCCCT-Q
NM_033217	NGFR
muNgfr - Forward	gTggAgAgTgCTgCAAAgC
muNgfr - Reverse	CgCTCACCACgTCAgAg
muNgfr -TM	F-CCTTgCggAgCCAACCAgACC-Q
NM_001025074	NRTK2
muNtrk2 - Forward	CTCAgCAAATCgCAgCAgg
muNtrk2 - Reverse	AgTAgTCggTgCTgTACA
muNtrk2 -TM	F-TTCACCAgCAggTTCTCTCCCACC-Q
NM_016894	RAMP1
muRAMP1- Forward	gTggggCTCTgCTTgC
muRAMP1-Reverse	gCACAgCTCCTggATgAgAgT
muRAMP1 -TM	F-ATCTCTTCATggTCACTgCCTgCCg-Q
NM_011703	VIPR1
muVipr1 - Forward	gCCTgTTCAggAAgCTgC
muVipr1 - Reverse	ggTCTgTCTCCCCgTTgT
muVipr1 -TM	F-CgAAACTACATCCACATgCATCTCTTCATg-Q
NM_01093	NPY1r
muNpy1r - Forward	CACgACTCTCCTCCTggTg
muNpy1r - Reverse	gCgAATgTATATCTTgAAgTAg
muNpy1r -TM	F-AgCAgAgTgggCCgAAATACTgCA-—Q
NM_009313	TACR1
Tacr1 - Forward	gACTTACgAgAAAgCgTACCACAT
Tacr1 - Reverse	CTggCCCACAgTgTAATCCCTA
Tacr1 -TM	FAM-TgTgTgACTgTgCTgATCTACTTCCTgCCT—BBQ

NM_007390	NAChRα7
nAChRα7 - Forward	GCCCTTGATAGCACAGTACTTCG
nAChRα7 - Reverse	GATCCTGGTCCACTTAGGCATTT
nAChRα7TM	6FAM-CAGTGGTCGTGACAGTGATTGTGCTGC-BBQ
NM_009311.2	TAC1
Tac1- Forward	CAACTGAGGAATCAGCATCC
Tac1 - Reverse	GCAGAGAATCGCCCGAA
Tac1-TM	6FAM-AgCCTCAgCAgTTCTTTggATTAATggg-BBQ
NM_007540	BDNF ex9
Bdnf- Forward	gTCTCTgCTTCCTTCCCACAg
Bdnf - Reverse	CCTTgTCCgTggACgTTTACT
TM	6FAM-CgCCTTCATgCAACCgAAgTATgAAA-BBQ
NM_ 009891.2	ChAT
Chat - Forward	CCAggACggTCCTCTTAAAAgAC
Chat - Reverse	CCCTgTgTgTgTCACTgAggT
Chat -TM	6FAM-CgggACTCCCTggACATgATCgAgC-BBQ
NM_ 013609.3	NGF
Ngf- Forward	gCAgTgAggTgCATAgCg
Ngf - Reverse	gTggAgTCTCCgTTTCTTAAACAgTC
Ngf-TM	6FAM-CCgCAgTgCCCCTACTgCACCAATA-–—BBQ
NM_011702	VIP
Vip - Forward	TgAATggAAAgAggAgCAgTg
Vip - Reverse	CgTggTTgTTTTCCTTCgAg
TM	6FAM- TTgAAgAgCTggAgAAATgATgggAAgA-–—BBQ
NM_023456	NPY
Npy - Forward	gCAgAggACATggCCAgATAC

Npy - Reverse	TgTgCTTTCCTTCATTAAgAggTC
TM	6FAM-CAAgAgATCCAgCCCTgAgACACTgA-—BBQ
NM_013684.3	TBP
Tbp - Forward	gTgAATCTTggCTgTAAACTTgACCT
Tbp - Reverse	gCAgTTgTCCgTggCTC
Tbf -TM	6FAM-AAATgCTgAATATAATCCCAAgCgATTTgC—BBQ
